# Dr. Elisha Townsend, of Philadelphia

**Published:** 1858-11

**Authors:** 


*Dr. Elisha Townsend, of Philadelphia.*—The death of this worthy gentleman will give pain to a large circle of friends; and by his death science has lost a most intelligent servant. He was president of the Dental College, Philadelphia, and has been instrumental in doing much to advance the true interests of the profession throughout the country. As a man, he possessed noble qualities; and as a friend, he was prompt, unselfish and sincere. He had recently visited Europe for his health, but returned only to die. He will long be remembered by those who knew him, and the absence of his aid and counsel will make a void not easily filled. E. G. T.

BOSTON, October 18, 1858.—*Boston Med. & Surg. Journal.*

